# Minimally invasive techniques for transthoracic oesophagectomy for oesophageal cancer: systematic review and network meta‐analysis

**DOI:** 10.1002/bjs5.50330

**Published:** 2020-09-07

**Authors:** K. Siaw‐Acheampong, S. K. Kamarajah, R. Gujjuri, J. R. Bundred, P. Singh, E. A. Griffiths

**Affiliations:** ^1^ College of Medical and Dental Sciences Newcastle upon Tyne UK; ^2^ Department of Hepatobiliary, Pancreatic and Transplant Surgery, Freeman Hospital Newcastle University NHS Foundation Trust Hospitals Newcastle upon Tyne UK; ^3^ Institute of Cellular Medicine University of Newcastle Newcastle upon Tyne UK; ^4^ Regional Oesophago‐Gastric Unit Royal Surrey County Hospital NHS Foundation Trust Guildford UK; ^5^ Institute of Cancer and Genomic Sciences, College of Medical and Dental Sciences University of Birmingham Birmingham UK; ^6^ Department of Upper Gastrointestinal Surgery University Hospitals Birmingham NHS Foundation Trust Birmingham UK

## Abstract

**Background:**

Oesophagectomy is a demanding operation that can be performed by different approaches including open surgery or a combination of minimal access techniques. This systematic review and network meta‐analysis aimed to evaluate the clinical outcomes of open, minimally invasive and robotic oesophagectomy techniques for oesophageal cancer.

**Methods:**

A systematic literature search was conducted for studies reporting open oesophagectomy, laparoscopically assisted oesophagectomy (LAO), thoracoscopically assisted oesophagectomy (TAO), totally minimally invasive oesophagectomy (MIO) or robotic MIO (RAMIO) for oesophagectomy. A network meta‐analysis of intraoperative (operating time, blood loss), postoperative (overall complications, anastomotic leaks, chyle leak, duration of hospital stay) and oncological (R0 resection, lymphadenectomy) outcomes, and survival was performed.

**Results:**

Ninety‐eight studies involving 32 315 patients were included in the network meta‐analysis (open 17 824, 55·2 per cent; LAO 1576, 4·9 per cent; TAO 2421 7·5 per cent; MIO 9558, 29·6 per cent; RAMIO 917, 2·8 per cent). Compared with open oesophagectomy, both MIO and RAMIO were associated with less blood loss, significantly lower rates of pulmonary complications, shorter duration of stay and higher lymph node yield. There were no significant differences between surgical techniques in surgical‐site infections, chyle leak, and 30‐ and 90‐day mortality. MIO and RAMIO had better 1‐ and 5‐year survival rates respectively compared with open surgery.

**Conclusion:**

Minimally invasive and robotic techniques for oesophagectomy are associated with reduced perioperative morbidity and duration of hospital stay, with no compromise of oncological outcomes but no improvement in perioperative mortality.

## Graphical abstract

Minimally invasive and robotic techniques for oesophagectomy are associated with reduced perioperative morbidity and length of hospital stay, with no compromise of oncological outcomes but no improvement in perioperative mortality. There are suggestions of improved long‐term survival with minimally invasive oesophagectomy and robotic minimally invasive oesophagectomy, but these associations are not consistent across 1‐, 3‐ and 5‐year survival.

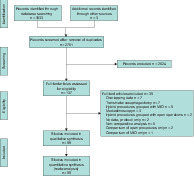

Support for move away from open surgery

## Abstract (es)

**Antecedentes:**

La esofaguectomía es una operación muy exigente que puede ser realizada a través de diferentes abordajes que incluyen la cirugía abierta o una combinación de técnicas con acceso mínimamente invasivo. Esta revisión sistemática y metaanálisis en red se propuso evaluar los resultados clínicos de la esofaguectomía abierta y de las técnicas de esofaguectomía mínimamente invasiva y robótica para el cáncer de esófago.

**Métodos:**

Se llevó a cabo una búsqueda sistemática de la bibliografía de estudios que describiesen esofaguectomía abierta, esofaguectomía asistida por laparoscopia (*laparoscopic assisted oesophagectomy*, LAO), esofaguectomía asistida por toracoscopia (*thoracoscopic assisted oesophagectomy*, TAO), esofaguectomía totalmente mínimamente invasiva (*totally minimally invasive oesophagectomy*, MIO) o MIO robótica (RAMIO). Se realizó un mataanálisis en red de resultados intraoperatorios (tiempo operatorio, pérdida de sangre), postoperatorios (complicaciones globales, fuga anastomótica, quilotórax, duración estancia hospitalaria), oncológicos (resección R0, linfadenectomía) y supervivencia.

**Resultados:**

Se incluyeron 98 estudios con 32.296 pacientes en el metaanálisis en red (abierta: *n* = 17.824, 55%; LAO: *n* = 1.576, 5%; TAO: *n* = 2.421, 7%; MIO: *n* = 9.558, 30%; RAMIO: *n* = 917, 3%). En comparación con la vía abierta, tanto MIO y RAMIO se asociaron con menos pérdidas hemáticas, tasas significativamente menores de complicaciones pulmonares, estancia más corta y obtención de un mayor número de ganglos linfáticos. No hubo diferencias significativas entre las técnicas quirúrgicas en las infecciones del sitio quirúrgico, quilotórax y mortalidad a los 30 y 90 días. MIO y RAMIO se asociaron con mejores tasas de supervivencia a 1 y 5 años respectivamente, en comparación con la cirugía abierta.

**Conclusión:**

Las técnicas mínimamente invasivas y robótica para la esofaguectomía se asociaron con menor morbilidad postoperatoria y estancia hospitalaria, sin comprometer los resultados oncológicos, pero sin mejoría en la mortalidad perioperatoria.

## Introduction

Oesophageal cancer remains a challenging disease worldwide, with over 570 000 new cases in 2018^1^. In managing this disease, oesophagectomy remains the mainstay of radical treatment with curative intent, with the transthoracic approach the most commonly employed. However, variation exists in surgical access techniques, with approximately 40 per cent of oesophagectomies in the UK now employing minimally invasive approaches^2^. The most common procedure is hybrid oesophagectomy where a laparoscopic gastric mobilization is performed with an open thoracotomy; a thoracoscopic–open abdominal hybrid procedure is uncommon. Less commonly both thoracoscopic and laparoscopic techniques are used in totally minimally invasive oesophagectomy (MIO). The use of robotic surgery for oesophagectomy is also increasing.

Since the development of minimally invasive approaches to oesophagectomy in the 1990s^3^, ^4^, ^5^, an evidence base has been growing to suggest similar, if not better, results in terms of morbidity and survival without compromising oncological benefit^6^, ^7^. This includes various pairwise meta‐analyses of mainly non‐randomized evidence^8^, ^9^, ^10^, ^11^, ^12^, ^13^, ^14^, ^15^, ^16^, ^17^, ^18^. Many of these studies grouped MIO together with hybrid procedures when comparing outcomes with those of open oesophagectomy.

Given the limited evidence and understanding of the potential benefits of different minimally invasive techniques for oesophagectomy, this systematic review and network meta‐analysis aimed to compare oncological safety and perioperative outcomes between these different surgical approaches and transthoracic oesophagectomy for cancer, along with impact on long‐term survival.

## Methods

### Search strategy

This study was conducted according to PRISMA guidelines^19^. A systematic and comprehensive search was undertaken of the MEDLINE, Embase and Cochrane Library databases, for studies published up to 25 February 2019. Search terms included the following, individually or in combination: ‘oesophagectomy’ or ‘oesophagectomy’ and ‘minimally invasive surgical procedures’ or ‘laparoscopy’ and ‘anastomotic leak’ or ‘postoperative complications’ or ‘lymph nodes examined’ or ‘survival’ and ‘oesophageal cancer’ or ‘esophageal cancer’. The full search strategy with all included search terms is shown in *Table S1* (supporting information). Manual scoping of reference lists in recent reviews was also undertaken. The protocol for this study was registered with the prospective PROSPERO database (CRD42019125848).

**Fig. 1:**
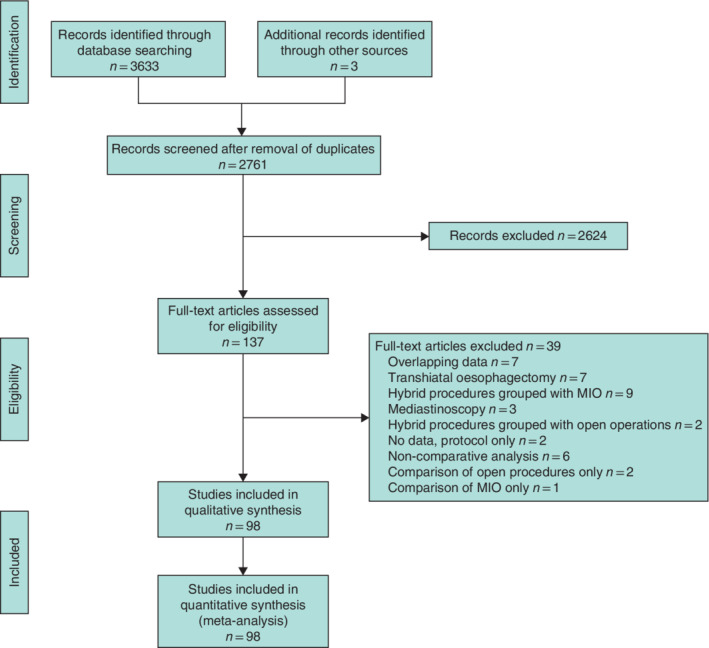
PRISMA diagram showing selection of articles for review MIO, minimally invasive oesophagectomy.

### Inclusion and exclusion criteria

Inclusion criteria were: comparative studies comparing any approach to two‐ or three‐stage transthoracic oesophagectomy in human subjects with cancer of the oesophagus or gastro‐oesophageal junction, and studies published in the English language. Exclusion criteria were: review articles; conference abstracts; studies with non‐comparative analyses of surgical approach including case reports; studies reporting transhiatal or left thoracoabdominal approaches; studies using a non‐gastric replacement conduit; and studies reporting pharyngolaryngoesophagectomy. After performing the literature search and removing all duplicates, two researchers screened the remaining titles and abstracts independently. Where a study was considered for inclusion, the full text was obtained. Discrepancies between the judgement of the two primary researchers were resolved through consensus with the other authors. Additionally, during full‐text review, authors of papers with mixed groups of both hybrid and totally minimally invasive techniques were contacted for separate data regarding each technique. Where multiple studies analysed the same data set or population, the most recent article was selected unless different outcomes were reported.

### Study outcomes

Outcome measures were: oncological – lymph node yield, R0 resection margins; intraoperative – blood loss and duration of operation; postoperative – duration of hospital stay, 30‐ and 90‐day mortality, overall, pulmonary, gastrointestinal and cardiac complications, anastomotic leak and chyle leak, and 1‐, 3‐ and 5‐year overall survival. The Esophageal Complications Consensus Group definitions of complications were used^20^. R0 status was defined using both College of American Pathologists^21^ and Royal College of Pathology^22^ definitions: absence of residual tumour at or within 1 mm of the resection margin respectively.

### Data extraction

Two researchers extracted data on study characteristics (author, year of publication, country, study interval, number of participants), patient characteristics (age, sex, BMI, overall TNM stage, location of anastomosis (cervical, thoracic), anastomotic technique (stapled *versus* handsewn), details of surgical approach and reported clinical outcomes.

### Definitions

Open oesophagectomy was defined as oesophagectomy carried out with laparotomy and open thoracotomy^23^, ^24^. MIO was defined as total MIO where laparoscopy was used for the abdominal phase and thoracoscopy for the thoracic phase. Laparoscopically assisted hybrid oesophagectomy (LAO) was defined as a laparoscopic abdominal phase combined with open thoracotomy. Thoracoscopically assisted hybrid oesophagectomy (TAO) was defined by an open abdominal phase combined with a thoracoscopic chest phase. Robotic MIO (RAMIO) was defined as oesophagectomy where either the abdominal or thoracic phase was performed using a robotic platform, including hybrid approaches^25^, ^26^. Regardless of access approach, two‐ and three‐stage oesophagectomies, with intrathoracic and cervical anastomoses respectively, were included, and a subgroup analysis was planned based on location of the anastomosis.

### Assessment of study quality

Methodological quality and standard of outcome reporting was assessed in each study by two independent researchers. Disagreements were settled through discussion between these researchers or consensus with all authors. For cohort studies, the Newcastle–Ottawa Scale^27^, ^28^ was used to formally assess quality, whereas the Cochrane risk‐of‐bias tool^29^ was used for RCTs.

### Statistical analysis

This systematic review and meta‐analysis was conducted in accordance with the recommendations of the Cochrane Library and PRISMA guidelines, as reported previously^30^. Dichotomous outcomes were compared using risk ratios (RRs), produced by meta‐analysis using random‐effects DerSimonian–Laird models. Heterogeneity between studies was assessed using the *I* ^2^ value, with values of less than 25, 25–75 and over 75 per cent considered to represent low, moderate and high degrees of heterogeneity respectively. Both randomized and non‐randomized studies were pooled into a network meta‐analysis comparing the above surgical approaches with transthoracic oesophagectomy. For each outcome, graphical representations of treatments (nodes) and comparisons (lines) were mapped. Network maps were then analysed for closed loops to be entered into network analyses.

Networks were examined for the presence of inconsistency, allowing for comparisons between direct and indirect treatment effects. Initially, this was assessed by checking for overall inconsistency throughout the entire network. A further check was then performed by fitting node side‐splitting models to identify loop inconsistency, within all three‐way treatment comparison loops, as described by Dias and colleagues^31^. If *P* exceeded 0·050, representing acceptance of the null hypothesis, consistency was assumed and networks were entered into consistency modelling. Consistency models used a restricted maximum likelihood model, generating network forest plots. Heterogeneity was examined by calculation of τ^2^. These were supplemented with interval plots of pooled effect estimates. Surgical approaches were then ranked using P*‐*scores, whereby a P‐score greater than 0·900 was considered to indicate the best technique with high probability. Subgroup analyses were conducted according to location of anastomoses, either cervical or thoracic, and for a more recent time cohort (2010 onwards). Statistical analyses for network meta‐analysis were undertaken using R version 3.2.1 (R Foundation for Statistical Computing, Vienna, Austria), with the netmeta packages, as described previously^32^, ^33^.

## Results

### Study characteristics

The review identified 98 studies^6^, ^7^, ^34^, ^35^, ^36^, ^37^, ^38^, ^39^, ^40^, ^41^, ^42^, ^43^, ^44^, ^45^, ^46^, ^47^, ^48^, ^49^, ^50^, ^51^, ^52^, ^53^, ^54^, ^55^, ^56^, ^57^, ^58^, ^59^, ^60^, ^61^, ^62^, ^63^, ^64^, ^65^, ^66^, ^67^, ^68^, ^69^, ^70^, ^71^, ^72^, ^73^, ^74^, ^75^, ^76^, ^77^, ^78^, ^79^, ^80^, ^81^, ^82^, ^83^, ^84^, ^85^, ^86^, ^87^, ^88^, ^89^, ^90^, ^91^, ^92^, ^93^, ^94^, ^95^, ^96^, ^97^, ^98^, ^99^, ^100^, ^101^, ^102^, ^103^, ^104^, ^105^, ^106^, ^107^, ^108^, ^109^, ^110^, ^111^, ^112^, ^113^, ^114^, ^115^, ^116^, ^117^, ^118^, ^119^, ^120^, ^121^, ^122^, ^123^, ^124^, ^125^, ^126^, ^127^, ^128^, ^129^ comparing surgical approaches for oesophagectomy, involving 32 315 patients (*Fig*. *1*). Of these, 55·2 per cent (17 824), 4·9 per cent (1576), 7·5 per cent (2421), 29·6 per cent (9558) and 2·8 per cent (917) were open oesophagectomy, LAO, TAO, MIO and RAMIO respectively. Study characteristics are presented in *Table* *1*. The majority of studies were non‐randomized (90). Eight studies were RCTs. Most studies compared two different oesophagectomy techniques; 14 compared at least three different techniques.

**Table 1:** Study‐ and patient‐level characteristics of articles included in review

						Anastomosis level		Anastomosis type			
Reference	Study design	Country	Comparison	No. of patients	Tumour location (U/M/L)	Cervical	Thoracic	Handsewn	Circular	Linear	Risk of bias/NOS score*
34	RCT	Austria	LAO *versus* open	26	n.r./n.r./n.r.	0	26	n.r.	n.r.	n.r.	Some concern
**7**	RCT	France	LAO *versus* open	207	3/63/141	0	207	n.r.	n.r.	n.r.	Low
35	RCT	China	MIO *versus* open	144	11/90/43	n.r.	n.r.	n.r.	n.r.	n.r.	High
36	RCT	Netherlands, Spain, Italy	MIO *versus* open	115	4/48/n.r.	75	32	n.r.	n.r.	n.r.	Low
6	RCT	Netherlands, Spain, Italy	MIO *versus* open	115	4/48/63	75	32	n.r.	n.r.	n.r.	Low
37	RCT	China	MIO *versus* open	114	0/0/0	114	0	114	0	0	High
38	RCT	China	MIO *versus* TAO	68	7/39/22	68	0	36	n.r.	n.r.	High
39	RCT	Netherlands	RAMIO *versus* open	109	1/13/55	106	0	106	0	0	Low
40	PCS	Serbia	LAO *versus* open	88	0/34/54	0	88	n.r.	n.r.	n.r.	7
41	PCS	UK	LAO *versus* open	70	n.r.	0	70	n.r.	n.r.	n.r.	6
42	PCS	UK	MIO *versus* LAO *versus* open	75	n.r.	0	75	26	n.r.	n.r.	6
43	PCS	UK	MIO *versus* LAO *versus* open	86	n.r.	n.r.	n.r.	n.r.	n.r.	n.r.	6
44	PCS	Sweden	MIO *versus* open	366	n.r.	261	105	n.r.	n.r.	n.r.	8
45	PCS	Taiwan	MIO *versus* open	190	15/91/83	190	0	99	53	38	8
46	PCS	UK	MIO *versus* open	106	0/4/46	0	106	1	0	105	5
47	PCS	Korea	MIO *versus* TAO	98	0/24/74	0	98	0	0	98	6
48	PCS	Japan	MIO *versus* TAO *versus* LAO *versus* open	210	26/133/51	198	12	n.r.	n.r.	n.r.	6
49	PCS	Australia	TAO *versus* open	487	0/43/355	n.r.	110	n.r.	n.r.	n.r.	8
50	PCS	Japan	TAO *versus* open	84	n.r.	n.r.	n.r.	n.r.	n.r.	n.r.	6
51	RCS	Japan	MIO *versus* TAO	315	n.r.	315	0	315	0	0	8
52	RCS	Japan	MIO *versus* TAO	64	6/14/44	64	0	n.r.	n.r.	n.r.	8
53	PCS	Germany	LAO *versus* MIO	60	n.r.	0	60	0	0	60	8
54	RCS	Sweden	LAO *versus* MIO	173	4/28/6	n.r.	n.r.	n.r.	n.r.	n.r.	6
55	RCS	Japan	LAO *versus* MIO	105	18/67/17	n.r.	n.r.	39	n.r.	n.r.	7
56	RCS	Japan	LAO *versus* open	216	41/108/67	216	0	0	0	216	8
57	RCS	South Korea	LAO *versus* open	115	n.r./36/79	0	115	3	4	108	7
58	RCS	China	LAO *versus* open	685	n.r.	0	685	n.r.	n.r.	n.r.	8
59	RCS	Germany	LAO *versus* open	120	0/16/104	0	120	n.r.	n.r.	n.r.	8
60	RCS	France	LAO *versus* open	140	0/123/17	0	140	n.r.	n.r.	n.r.	8
61	RCS	France	LAO *versus* open	280	0/110/170	0	280	n.r.	n.r.	n.r.	8
62	RCS	Italy	LAO *versus* open	68	n.r.	13	55	n.r.	n.r.	n.r.	8
63	RCS	UK	MIO *versus* LAO *versus* open	334	0/22/122	n.r.	n.r.	67	n.r.	n.r.	8
64	RCS	China	MIO *versus* LAO/TAO *versus* open	548	154/331/63	548	0	n.r.	n.r.	n.r.	6
65	RCS	Pakistan	MIO *versus* LAO/TAO *versus* open	216	n.r.	n.r.	n.r.	n.r.	n.r.	n.r.	8
66	RCS	Japan	MIO *versus* open	98	8/60/30	9	89	12	0	84	7
67	RCS	Japan	MIO *versus* open	171	3/44/45	171	0	0	0	171	7
68	RCS	Japan	MIO *versus* open	130	n.r.	65	65	n.r.	n.r.	n.r.	8
69	RCS	China	MIO *versus* open	63	n.r.	0	63	n.r.	n.r.	n.r.	8
70	RCS	China	MIO *versus* open	228	3/130/95	n.r.	n.r.	n.r.	n.r.	n.r.	7
71	RCS	China	MIO *versus* open	269	0/191/78	0	269	n.r.	n.r.	n.r.	7
72	RCS	China	MIO *versus* open	221	20/154/47	n.r.	n.r.	n.r.	n.r.	n.r.	7
73	RCS	USA	MIO *versus* open	39	n.r.	39	0	n.r.	n.r.	n.r.	6
74	RCS	China	MIO *versus* open	257	54/169/34	257	0	62	0	195	8
75	RCS	Netherlands	MIO *versus* open	866	16/189/517	563	303	n.r.	n.r.	n.r.	8
76	RCS	China	MIO *versus* open	183	24/118/41	183	0	n.r.	n.r.	n.r.	7
77	RCS	China	MIO *versus* open	80	7/56/17	80	0	0	80	0	6
78	RCS	Finland	MIO *versus* open	153	n.r.	0	153	79	0	73	7
79	RCS	USA	MIO *versus* open	168	n.r.	n.r.	n.r.	n.r.	n.r.	n.r.	6
80	RCS	USA	MIO *versus* open	130	0/5/72	n.r.	n.r.	n.r.	n.r.	n.r.	7
81	RCS	USA	MIO *versus* open	114	n.r.	0	114	0	0	114	8
82	RCS	Japan	MIO *versus* open	62	9/34/9	62	0	n.r.	n.r.	n.r.	7
83	RCS	China	MIO *versus* open	113	0/113/0	113	0	0	0	113	6
84	RCS	China	MIO *versus* open	230	94/115/21	230	0	n.r.	n.r.	n.r.	7
85	RCS	Finland, Sweden	MIO *versus* open	1614	n.r.	n.r.	n.r.	n.r.	n.r.	n.r.	6
86	RCS	USA	MIO *versus* open	146	0/3/0	138	8	n.r.	n.r.	n.r.	8
87	RCS	UK	MIO *versus* open	80	n.r./n.r./10	49	31	n.r.	n.r.	n.r.	6
88	RCS	China	MIO *versus* open	379	n.r.	0	379	0	0	379	7
89	RCS	China	MIO *versus* open	118	7/74/37	118	0	n.r.	n.r.	n.r.	8
90	RCS	China	MIO *versus* open	447	n.r.	348	99	n.r.	n.r.	n.r.	7
91	RCS	Netherlands, Spain, Italy	MIO *versus* open	575	n.r.	n.r.	n.r.	n.r.	n.r.	n.r.	7
92	RCS	USA	MIO *versus* open	4047	n.r.	n.r.	n.r.	n.r.	n.r.	n.r.	8
93	RCS	China	MIO *versus* open	118	0/49/69	n.r.	n.r.	n.r.	n.r.	n.r.	7
94	RCS	China	MIO *versus* open	194	35/87/72	n.r.	n.r.	n.r.	n.r.	n.r.	8
95	RCS	UK	MIO *versus* open	7502	n.r.	n.r.	n.r.	n.r.	n.r.	n.r.	8
96	RCS	Belgium	MIO *versus* open	166	n.r.	166	0	n.r.	n.r.	n.r.	7
97	RCS	Japan	MIO *versus* open	92	6/60/26	92	0	n.r.	n.r.	n.r.	8
98	RCS	China	MIO *versus* open	174	15/127/32	174	0	n.r.	n.r.	n.r.	7
99	RCS	China	MIO *versus* open	162	n.r.	n.r.	n.r.	n.r.	n.r.	n.r.	7
100	RCS	China	MIO *versus* open	407	25/290/92	n.r.	n.r.	n.r.	n.r.	n.r.	7
101	RCS	China	MIO *versus* TAO	172	54/73/45	172	0	n.r.	n.r.	n.r.	8
102	RCS	Japan	MIO *versus* TAO	64	7/23/34	64	0	64	0	0	6
103	RCS	Italy	MIO *versus* TAO	160	6/29/125	80	80	0	160	0	8
104	RCS	China	MIO *versus* TAO *versus* LAO *versus* open	109	16/59/34	n.r.	n.r.	n.r.	n.r.	n.r.	7
105	RCS	Japan	MIO *versus* TAO *versus* open	242	36/137/69	242	0	n.r.	n.r.	n.r.	8
106	RCS	Japan	MIO *versus* TAO *versus* open	185	33/85/67	170	15	97	0	88	7
107	RCS	China	MIO *versus* TAO *versus* open	138	23/n.r./n.r.	138	0	n.r.	n.r.	n.r.	6
108	RCS	Thailand	MIO *versus* TAO *versus* open	83	17/41/25	83	0	n.r.	n.r.	n.r.	7
109	RCS	Australia	MIO *versus* TAO *versus* open	446	10/84/262	n.r.	n.r.	n.r.	n.r.	n.r.	6
110	RCS	Australia	MIO *versus* TAO *versus* open	858	15/78/524	858	0	n.r.	n.r.	n.r.	7
111	RCS	Taiwan	RAMIO *versus* MIO	68	20/34/14	68	0	n.r.	n.r.	n.r.	8
112	RCS	South Korea	RAMIO *versus* MIO	105	15/24/66	56	35	n.r.	n.r.	n.r.	6
113	RCS	China	RAMIO *versus* MIO	54	4/33/n.r.	54	0	0	0	54	8
114	RCS	USA	RAMIO *versus* MIO	37	n.r.	37	0	0	24	0	7
115	RCS	China	RAMIO *versus* MIO	84	0/84/0	84	0	84	0	0	7
116	RCS	USA	RAMIO *versus* MIO *versus* open	1707	n.r.	n.r.	n.r.	n.r.	n.r.	n.r.	8
117	RCS	South Korea	RAMIO *versus* open	247	n.r.	247	0	n.r.	n.r.	n.r.	8
118	RCS	Japan	RAMIO *versus* open	60	2/30/28	n.r.	n.r.	n.r.	n.r.	n.r.	7
119	RCS	China	TAO *versus* open	78	9/48/21	78	0	n.r.	n.r.	n.r.	7
120	RCS	China	TAO *versus* open	108	20/88/0	n.r.	n.r.	n.r.	n.r.	n.r.	7
121	RCS	Japan	TAO *versus* open	257	32/143/82	n.r.	n.r.	n.r.	n.r.	n.r.	9
122	RCS	Japan	TAO *versus* open	59	9/31/19	59	0	59	0	0	5
123	RCS	South Korea	TAO *versus* open	84	n.r./61/23	14	70	0	0	84	8
124	RCS	Japan	TAO *versus* open	51	5/34/12	51	0	n.r.	n.r.	n.r.	6
125	RCS	Japan	TAO *versus* open	149	23/85/41	149	0	n.r.	n.r.	n.r.	7
126	RCS	Taiwan	TAO *versus* open	129	20/63/36	129	0	n.r.	n.r.	n.r.	6
127	RCS	Japan	TAO *versus* open	329	52/193/84	n.r.	n.r.	n.r.	n.r.	n.r.	7
128	RCS	Hong Kong	TAO *versus* open	81	8/61/9	18	63	18	n.r.	n.r.	7
129	RCS	China	TAO *versus* open	178	26/68/84	n.r.	n.r.	n.r.	n.r.	n.r.	8

* For RCTs, the risk of bias was determined as low, high or of some concern. U, upper; M, middle; L, lower; NOS, Newcastle–Ottawa Scale; LAO, laparoscopically assisted oesophagectomy; n.r., not reported; MIO, minimally invasive oesophagectomy; TAO, thoracoscopically assisted oesophagectomy; RAMIO, robotic minimally invasive oesophagectomy; PCS, prospective cohort study; RCS, retrospective cohort study.

Studies involving open oesophagectomy (82) and MIO (71) were the most commonly reported. TAO was analysed in 30 studies, of which 13 compared it with open oesophagectomy only, seven with MIO only, and ten with both open and MIO. LAO was compared with open surgery in 12 articles, whereas three analysed it against MIO. There were eight papers comparing RAMIO with MIO (5) and open oesophagectomy (3). Subgroup analyses by location of anastomoses are presented in *Tables S2–S5* (supporting information).

### Intraoperative outcomes

*Table* *2* shows the results of pairwise comparisons between intraoperative outcomes, and network maps are presented in *Fig. S1* (supporting information). Duration of operation was reported in 77 studies. Open surgery resulted in significantly shorter operating times than MIO (mean difference (MD) 37 min; *P* < 0·001), RAMIO (MD 75 min; *P* < 0·001) and TAO (MD 21 min; *P* = 0·011) (*Table* *2*). Open surgery had the shortest operating time, with a high probability, followed by hybrid operations then MIO and RAMIO (*Table* *3*). Open oesophagectomy was ranked first for cervical anastomosis, whereas LAO was ranked first for thoracic anastomosis (*Tables S2* and *S3*, supporting information).

**Table 2:** Summary of intraoperative outcomes of overall network meta‐analysis

	Duration of surgery (min)			Blood loss (ml)		
	No. of studies	Mean difference	*P*	No. of studies	Mean difference	*P*
Open *versus* TAO	15	–21 (–37, –5)	0·011	16	91 (49, 133)	< 0·001
Open *versus* LAO	13	0 (–19, 19)	0·997	4	84 (16, 153)	0·016
Open *versus* MIO	37	–37 (–48, –26)	< 0·001	36	173 (146, 200)	< 0·001
Open *versus* RAMIO	3	–75 (–104, –46)	< 0·001	3	163 (99, 226)	< 0·001
MIO *versus* TAO	12	16 (–1, 33)	0·063	12	–82 (–125, –39)	< 0·001
MIO *versus* LAO	5	37 (16, 58)	< 0·001	4	–88 (–158, –20)	0·012
MIO *versus* RAMIO	4	–38 (–67, –9)	0·011	5	–10 (–73, 52)	0·750
LAO *versus* TAO	1	–21 (–45, 3)	0·090	1	7 (–71, 85)	0·867
RAMIO *versus* TAO	0	54 (21, 86)	0·001	0	–72 (–145, 2)	0·056
RAMIO *versus* LAO	0	75 (40, 109)	< 0·001	0	–78 (–170, 13)	0·093

Values in parentheses are 95 per cent confidence intervals. TAO, thoracoscopically assisted oesophagectomy; LAO, laparoscopically assisted oesophagectomy; MIO, minimally invasive oesophagectomy; RAMIO, robotic minimally invasive oesophagectomy.

**Table 3:** Ranking of surgical techniques for intraoperative, oncological and postoperative outcomes according to P*‐*scores

	Rank				
	1	2	3	4	5
Duration of operation	Open (P = 0·874)	LAO (P = 0·863)	TAO (P = 0·505)	MIO (P = 0·257)	RAMIO (P = 0·002)
Blood loss	MIO (P = 0·905)	RAMIO (P = 0·825)	TAO (P = 0·399)	LAO (P = 0·369)	Open (P = 0·002)
Overall complications	RAMIO (P = 0·872)	LAO (P = 0·672)	MIO (P = 0·657)	TAO (P = 0·199)	Open (P = 0·101)
Pulmonary complications	MIO (P = 0·872)	TAO (P = 0·632)	RAMIO (P = 0·550)	LAO (P = 0·414)	Open (P = 0·031)
Cardiac complications	RAMIO (P = 0·987)	LAO (P = 0·688)	MIO (P = 0·548)	Open (P = 0·219)	TAO (P = 0·058)
Anastomotic leak	TAO (P = 0·810)	MIO (P = 0·775)	Open (P = 0·443)	RAMIO (P = 0·367)	LAO (P = 0·106)
Wound/diaphragm complications	TAO (P = 0·885)	RAMIO (P = 0·661)	Open (P = 0·434)	MIO (P = 0·295)	LAO (P = 0·226)
Gastrointestinal complications	MIO (P = 0·854)	TAO (P = 0·684)	Open (P = 0·478)	RAMIO (P = 0·347)	LAO (P = 0·136)
Chyle leak	LAO (P = 0·704)	Open (P = 0·659)	MIO (P = 0·558)	TAO (P = 0·332)	RAMIO (P = 0·247)
Duration of hospital stay	RAMIO (P = 0·911)	MIO (P = 0·707)	TAO (P = 0·625)	LAO (P = 0·229)	Open (P = 0·028)
30‐day mortality	Open (P = 0·697)	MIO (P = 0·562)	LAO (P = 0·538)	TAO (P = 0·368)	RAMIO (P = 0·334)
90‐day mortality	LAO (P = 0·779)	MIO (P = 0·541)	Open (P = 0·417)	RAMIO (P = 0·264)	–
Lymph nodes examined	RAMIO (P = 0·969)	MIO (P = 0·698)	TAO (P = 0·418)	Open (P = 0·253)	LAO (P = 0·162)
R0 resection	RAMIO (P = 0·729)	MIO (P = 0·699)	TAO (P = 0·629)	LAO (P = 0·315)	Open (P = 0·129)
1‐year survival	TAO (P = 0·861)	MIO (P = 0·682)	LAO (P = 0·544)	Open (P = 0·218)	RAMIO (P = 0·194)
3‐year survival	TAO (P = 0·751)	LAO (P = 0·551)	RAMIO (P = 0·544)	MIO (P = 0·436)	Open (P = 0·219)
5‐year survival	RAMIO (P = 0·949)	TAO (P = 0·609)	MIO (P = 0·506)	LAO (P = 0·360)	Open (P = 0·076)

LAO, laparoscopically assisted oesophagectomy; TAO, thoracoscopically assisted oesophagectomy; MIO, minimally invasive oesophagectomy; RAMIO, robotic minimally invasive oesophagectomy.

Blood loss was reported in 65 studies. Open oesophagectomy had significantly higher blood loss than TAO (MD 91 ml; *P* < 0·001), LAO (MD 84 ml; *P* = 0·016), RAMIO (MD 163 ml; *P* < 0·001) and MIO (MD 173 ml; *P =* 0·001). MIO was ranked first for lowest blood loss, with a high probability, followed by RAMIO (*Table* *3*). MIO was ranked first for both cervical and thoracic anastomosis, followed by RAMIO (*Tables S2* and *S3*, supporting information).

### Postoperative outcomes

The results of all pairwise comparisons of each surgical approach for postoperative complications are shown in *Tables* *4* and *5*, and network maps in *Fig. S2* (supporting information). There were no significant differences between surgical approaches for surgical‐site infections, chyle leak and 30‐ or 90‐day mortality.

**Table 4:** Summary of postoperative complications in overall network meta‐analysis

	No. of studies	Risk ratio	*P*	No. of studies	Risk ratio	*P*	No. of studies	Risk ratio	*P*
	**Overall complications**			**Pulmonary complications**			**Cardiac complications**		
Open *versus* TAO	6	1·07 (0·72, 1·58)	0·742	15	1·66 (1·17, 2·35)	0·004	7	0·86 (0·62, 1·19)	0·356
Open *versus* LAO	9	1·59 (1·11, 2·22)	0·010	15	1·39 (0·97, 2·00)	0·073	8	1·41 (0·95, 2·08)	0·089
Open *versus* MIO	17	1·54 (1·22, 1·96)	< 0·001	40	1·92 (1·54, 2·38)	< 0·001	28	1·19 (1·03, 1·37)	0·015
Open *versus* RAMIO	1	2·20 (0·98, 4·97)	0·057	3	0·87 (0·82, 0·92)	0·001	2	2·87 (1·43, 5·75)	0·003
MIO *versus* TAO	3	0·69 (0·46, 1·04)	0·079	11	0·86 (0·60, 1·24)	0·415	6	0·72 (0·52, 1·00)	0·051
MIO *versus* LAO	2	1·02 (0·68, 1·52)	0·931	7	0·72 (0·49, 1·09)	0·110	2	1·18 (0·78, 1·79)	0·431
MIO *versus* RAMIO	1	1·42 (0·63, 3·24)	0·401	5	0·81 (0·43, 1·51)	0·498	2	2·41 (1·19, 4·89)	0·015
LAO *versus* TAO	0	0·68 (0·40, 1·14)	0·139	1	1·19 (0·72, 1·94)	0·498	0	0·61 (0·37, 1·02)	0·057
RAMIO *versus* TAO	0	0·49 (0·20, 1·19)	0·112	0	1·07 (0·53, 2·17)	0·854	0	0·30 (0·14, 0·64)	0·002
RAMIO *versus* LAO	0	0·71 (0·30, 1·72)	0·457	0	0·90 (0·44, 1·85)	0·777	0	0·49 (0·22, 1·09)	0·081
	**Anastomotic leak**			**Surgical‐site infection**			**Gastrointestinal complications**		
Open *versus* TAO	15	1·22 (0·88, 1·73)	0·237	1	6·09 (0·82, 45·06)	0·077	15	1·11 (0·79, 1·56)	0·544
Open *versus* LAO	14	0·72 (0·47, 1·11)	0·136	5	0·70 (0·23, 2·17)	0·540	15	0·75 (0·51, 1·11)	0·160
Open *versus* MIO	39	1·18 (0·93, 1·49)	0·170	12	0·83 (0·43, 1·64)	0·599	41	1·20 (0·96, 1·49)	0·109
Open *versus* RAMIO	3	0·88 (0·44, 1·79)	0·730	1	3·00 (0·10, 94·13)	0·532	3	0·85 (0·40, 1·79)	0·670
MIO *versus* TAO	13	1·04 (0·74, 1·46)	0·829	1	7·29 (0·95, 56·01)	0·056	13	0·93 (0·66, 1·31)	0·663
MIO *versus* LAO	8	0·61 (0·39, 0·95)	0·030	2	0·84 (0·27, 2·63)	0·771	9	0·63 (0·42, 0·94)	0·024
MIO *versus* RAMIO	5	0·75 (0·37, 1·54)	0·430	0	3·59 (0·11, 120·31)	0·475	5	0·71 (0·34, 1·50)	0·369
LAO *versus* TAO	2	1·69 (1·01, 2·86)	0·048	0	8·66 (0·90, 83·54)	0·062	0	1·47 (0·90, 2·42)	0·127
RAMIO *versus* TAO	0	1·39 (0·64, 3·00)	0·406	0	2·03 (0·04, 109·19)	0·728	0	1·31 (0·58, 2·93)	0·517
RAMIO *versus* LAO	0	0·82 (0·36, 1·85)	0·631	0	0·23 (0·01, 9·09)	0·433	2	0·88 (0·39, 2·04)	0·778
	**Chyle leak**			**Duration of hospital stay (days)**					
Open *versus* TAO	9	0·81 (0·49, 1·32)	0·391	13	2·77 (1·60, 3·93)*	< 0·001			
Open *versus* LAO	7	1·12 (0·51, 2·44)	0·780	12	0·87 (0·53, 2·26)*	0·223			
Open *versus* MIO	22	0·95 (0·71, 1·28)	0·750	38	3·00 (2·30, 3·70)*	< 0·001			
Open *versus* RAMIO	1	0·69 (0·31, 1·54)	0·368	2	3·85 (1·80, 5·71)*	< 0·001			
MIO *versus* TAO	5	0·84 (0·50, 1·43)	0·531	12	–0·23 (–1·43, 1·00)*	0·706			
MIO *versus* LAO	3	1·18 (0·52, 2·63)	0·700	4	–2·13 (–3·64, –0·63)*	0·005			
MIO *versus* RAMIO	2	0·73 (0·32, 1·68)	0·454	3	0·85 (–1·01, 2·70)*	0·371			
LAO *versus* TAO	0	0·72 (0·29, 1·81)	0·485	1	1·90 (0·12, 3·69)*	0·036			
RAMIO *versus* TAO	0	1·16 (0·46, 2·96)	0·754	0	–1·08 (–3·23, 1·07)*	0·326			
RAMIO *versus* LAO	0	1·61 (0·53, 5·00)	0·402	0	–2·98 (–5·29, –0·67)*	0·011			

Values in parentheses are 95 per cent confidence intervals.

* Mean difference. TAO, thoracoscopically assisted oesophagectomy; LAO, laparoscopically assisted oesophagectomy; MIO, minimally invasive oesophagectomy; RAMIO, robotic minimally invasive oesophagectomy.

**Table 5:** Summary of postoperative mortality and survival in overall network meta‐analysis

	No. of studies	Risk ratio	*P*	No. of studies	Risk ratio	*P*	No. of studies	Risk ratio	*P*
	**30‐day mortality**			**90‐day mortality**			**1‐year survival**		
Open *versus* TAO	2	0·62 (0·14, 2·67)	0·517				9	1·62 (1·01, 2·58)	0·043
Open *versus* LAO	7	0·84 (0·27, 2·63)	0·768	0	1·47 (0·58, 3·70)	0·410	9	1·23 (0·79, 1·92)	0·361
Open *versus* MIO	20	0·87 (0·46, 1·64)	0·672	5	1·08 (0·66, 1·75)	0·772	26	1·35 (1·02, 1·79)	0·035
Open *versus* RAMIO	2	0·57 (0·12, 2·61)	0·469	2	0·87 (0·45, 1·70)	0·683	2	0·86 (0·40, 1·86)	0·714
MIO *versus* TAO	3	0·71 (0·17, 2·94)	0·634				2	1·20 (0·71, 2·03)	0·506
MIO *versus* LAO	2	0·97 (0·28, 3·33)	0·958	0	1·37 (0·48, 4·00)	0·552	3	0·92 (0·56, 1·52)	0·749
MIO *versus* RAMIO	3	0·65 (0·15, 2·96)	0·582	3	0·81 (0·42, 1·58)	0·535	2	0·64 (0·29, 1·39)	0·267
LAO *versus* TAO	0	0·73 (0·12, 4·52)	0·736				0	1·31 (0·69, 2·51)	0·420
RAMIO *versus* TAO	0	1·08 (0·14, 8·34)	0·940				0	1·88 (0·77, 4·61)	0·167
RAMIO *versus* LAO	0	1·47 (0·23, 10·00)	0·682	0	1·69 (0·54, 5·26)	0·364	0	1·43 (0·50, 3·45)	0·436
	**3‐year survival**			**5‐year survival**					
Open *versus* TAO	8	1·38 (0·86, 2·22)	0·184	7	1·49 (0·94, 2·34)	0·086			
Open *versus* LAO	8	1·19 (0·76, 1·85)	0·453	5	1·20 (0·76, 1·89)	0·428			
Open *versus* MIO	23	1·10 (0·83, 1·45)	0·514	18	1·33 (1·00, 1·79)	0·051			
Open *versus* RAMIO	2	1·20 (0·58, 2·48)	0·636	0	4·00 (1·05, 15·33)	0·042			
MIO *versus* TAO	1	1·26 (0·73, 2·15)	0·409	1	1·11 (0·66, 1·89)	0·711			
MIO *versus* LAO	2	1·08 (0·65, 1·79)	0·790	2	0·90 (0·55, 1·49)	0·697			
MIO *versus* RAMIO	2	1·09 (0·53, 2·26)	0·827	1	3·00 (0·81, 11·12)	0·100			
LAO *versus* TAO	0	1·16 (0·61, 2·23)	0·667	0	1·23 (0·65, 2·35)	0·539			
RAMIO *versus* TAO	0	1·15 (0·48, 2·72)	0·765	0	0·37 (0·09, 1·53)	0·170			
RAMIO *versus* LAO	0	0·99 (0·42, 2·27)	0·983	0	0·30 (0·07, 1·22)	0·093			

Values in parentheses are 95 per cent confidence intervals. TAO, thoracoscopically assisted oesophagectomy; LAO, laparoscopically assisted oesophagectomy; MIO, minimally invasive oesophagectomy; RAMIO, robotic minimally invasive oesophagectomy.

#### Overall complications

Overall complications were reported in 39 studies. LAO (RR 0·63; *P =* 0·010) and MIO (RR 0·65; *P* < 0·001) had significantly lower rates of overall complications than open surgery (*Table* *4*). RAMIO was ranked best for overall complications (*Table* *3*). MIO was ranked first for cervical anastomosis, whereas RAMIO was ranked first for thoracic anastomosis (*Tables S3* and *S4*, supporting information).

#### Pulmonary complications

Pulmonary complications were reported in 79 studies. MIO (RR 0·52; *P* < 0·001) and TAO (RR 0·60; *P =* 0·004) were associated with significantly lower rates of pulmonary complications than open surgery. MIO was ranked the best technique in terms of pulmonary complications overall (*Table* *3*), and in subgroups of cervical and thoracic anastomoses (*Tables S3* and *S4*, supporting information).

#### Cardiac complications

Cardiac complications were reported in 46 studies. RAMIO (RR 0·35; *P =* 0·003) and MIO (RR 0·84; *P =* 0·015) were associated with significantly lower rates of cardiac complications than open oesophagectomy. RAMIO had significantly lower rates of cardiac complications compared with TAO (RR 0·30; *P =* 0·002) and MIO (RR 0·42; *P =* 0·015). RAMIO was ranked first for cardiac complications (*Table* *3*).

#### Anastomotic leaks

Anastomotic leak was reported in 74 studies. LAO was significantly associated with a higher rate of anastomotic leak than TAO (RR 1·69; *P =* 0·048) and MIO (RR 1·63; *P =* 0·030) (*Table* *4*). TAO was ranked first for anastomotic leak (*Table* *3*). In terms of anastomotic leakage, TAO was ranked first for thoracic anastomosis, whereas RAMIO was ranked first for cervical anastomosis (*Tables S3* and *S4*, supporting information).

#### Duration of hospital stay

Length of hospital stay was reported in 72 studies. MIO (MD 3·00 days; *P* < 0·001), RAMIO (MD 3·85 days; *P* < 0·001) and TAO (MD 2·77 days; *P* < 0·001) were associated with significantly shorter duration of stay compared with open oesophagectomy. RAMIO (MD 2·98 days; *P =* 0·011) and TAO (MD 1·90 days; *P =* 0·036) were also associated with significantly shorter hospital stay than LAO. RAMIO was ranked first, with a high probability, followed by MIO (*Table* *3*).

#### Overall survival

One‐year overall survival was reported in 53 studies. The open approach was associated with significantly lower 1‐year survival than TAO (RR 1·62; *P =* 0·043) and MIO (RR 1·35; *P =* 0·035) (*Table* 5). Overall, TAO was ranked first for 1‐year survival (*Table* *3*). However, MIO and LAO were ranked first for cervical and thoracic anastomosis respectively (*Tables S3* and *S4*, supporting information). Three‐year overall survival was reported in 46 studies. There were no significant differences in outcomes between any techniques. Five‐year overall survival was reported in 34 studies. Open oesophagectomy was associated with significantly lower 5‐year survival than RAMIO (RR 4·00; *P =* 0·042) (*Table* *5*). Overall, RAMIO was ranked the best technique, with high probability (*Table* *3*). A sensitivity analysis for 1‐ and 5‐year survival including studies from 2010 onwards yielded similar results.

### Oncological outcomes

#### Lymph nodes examined

The results of all pairwise comparisons of oncological outcomes for each surgical approach technique are shown in *Table* *6*, and network maps in *Fig. S3* (supporting information). Lymph node assessment was reported in 77 studies. LAO (mean difference 3·53; *P =* 0·031) and open surgery (mean difference 3·11; *P =* 0·024) were associated with significantly lower numbers of lymph nodes examined than RAMIO. RAMIO was ranked as the best technique, with high probability, followed by MIO (*Table* *3*).

**Table 6:** Summary of oncological outcomes of overall network meta‐analysis

	Lymph nodes examined			Negative resection margins (R0)		
Comparison	No. of studies	Mean difference	*P*	No. of studies	Risk ratio	*P*
Open *versus* TAO	17	–0·37 (–1·79, 1·05)	0·606	6	0·75 (0·51, 1·11)	0·150
Open *versus* LAO	11	0·42 (–1·35, 2·20)	0·640	7	0·93 (0·57, 1·49)	0·756
Open *versus* MIO	34	–1·06 (–2·05, –0·08)	0·035	14	0·73 (0·60, 0·89)	0·002
Open *versus* RAMIO	2	–3·11 (–5·80, –0·41)	0·024	1	0·70 (0·44, 1·10)	0·121
MIO *versus* TAO	11	0·69 (–0·82, 2·20)	0·370	4	1·03 (0·70, 1·53)	0·885
MIO *versus* LAO	3	1·49 (0·49, 3·46)	0·014	0	1·27 (0·75, 2·13)	0·369
MIO *versus* RAMIO	6	–2·04 (–4·65, 0·57)	0·125	2	0·96 (0·60, 1·51)	0·844
LAO *versus* TAO	1	–0·80 (–3·04, 1·45)	0·487	0	0·81 (0·44, 1·50)	0·505
RAMIO *versus* TAO	0	2·73 (–0·23, 5·69)	0·070	0	1·08 (0·60, 1·93)	0·801
RAMIO *versus* LAO	0	3·53 (0·33, 6·73)	0·031	0	1·33 (0·68, 2·56)	0·399

Values in parentheses are 95 per cent confidence intervals. TAO, thoracoscopically assisted oesophagectomy; LAO, laparoscopically assisted oesophagectomy; MIO, minimally invasive oesophagectomy; RAMIO, robotic minimally invasive oesophagectomy.

#### R0 resection

R0 resections were reported in 40 studies. MIO was associated with higher rates of R0 resection (RR 1·37; *P =* 0·002) than open surgery. RAMIO was ranked first, followed by MIO (*Table* *3*).

## Discussion

This network meta‐analysis compared all combinations of open, minimally invasive and robotic approaches to transthoracic oesophagectomy. The analysis demonstrated that minimally invasive surgery for oesophagectomy was associated with increased operating time, but decreased operative blood loss, fewer pulmonary complications and shorter length of hospital stay, compared with open approaches. In addition, the review identified significantly decreased overall postoperative complications with minimally invasive surgery compared with the open approach. Importantly, no significant differences in perioperative mortality (either 30 or 90 day) were observed between any surgical approach. In addition, MIO and RAMIO were associated with significantly higher 1‐ and 5‐year survival rates respectively than open oesophagectomy. These findings were not altered in a sensitivity analysis including studies from 2010 onwards. Based on the present evidence, no one approach demonstrates clear overall superiority over all others, but there is increasing evidence of the specific benefits related to minimally invasive techniques.

Network meta‐analysis allows assessment of different surgical techniques by combining direct evidence within studies and indirect evidence across studies. Hence, it enables indirect comparisons of surgical techniques that have not been studied directly in a head‐to‐head fashion^130^. By including evidence from both direct and indirect comparisons, a network meta‐analysis may increase the precision in estimates of the relative effects of treatments and improve power compared with standard pairwise meta‐analyses that include only direct evidence^131^. Network meta‐analysis may yield more reliable and definitive results, and allows visualization and interpretation of a wider picture of the available evidence, and to calculate treatment rankings with probabilities, compared with a standard pairwise meta‐analysis^130^.

This study has some limitations. The majority of the studies included in this network meta‐analysis subject it to heterogeneity owing to patient selection criteria and demographics, such as age, sex, BMI and different disease stages. The amount of evidence a treatment carries and the number of comparisons available between treatments determines the diversity and strength of a network meta‐analysis. Imbalance in terms of the amount of evidence available may affect the power and reliability of the network meta‐analysis as inferences may be driven largely from the evidence from few treatments and comparisons^132^. Some of the studies assessed new techniques or technologies and may have incorporated a learning curve in the novel arm.

Previous standard pairwise meta‐analyses^8^, ^9^, ^10^, ^11^, ^12^, ^13^, ^14^, ^15^, ^16^, ^17^, ^18^ and RCTs^6^, ^7^, ^34^, ^35^ comparing open *versus* minimally invasive resection for oesophagectomy demonstrated that, although laparoscopic surgery increased operative time, it resulted in significantly reduced blood loss and wound infection, increased R0 resection rate and shorter hospital stay. In addition, the present review identified significantly decreased overall postoperative complications with minimal access compared with open surgery, and this may be related to the lower wound infection rate and pulmonary complications of the minimally invasive approach.

This network meta‐analysis identified that minimally invasive surgery was associated with significantly more examined lymph nodes compared with open surgery, specifically with RAMIO and MIO techniques. Evidence from RCTs^6^, ^7^ is limited as none have demonstrated the superiority of either laparoscopic or open techniques. This network meta‐analysis also showed that rates of R0 resection were better with MIO compared with open surgery. This is an important point as one of the barriers to adoption of the minimally invasive approach in routine clinical practice over conventional open oesophagectomy was concern over oncological clearance as R0 resections are recognized to be an important prognostic marker of long‐term survival following surgery^133^, ^134^. It is also important to note that differences in R0 resection rates may also be attributed to differences in the R0 classification systems used.

Both RAMIO and MIO techniques were associated with significantly lower rates of pulmonary complications and shorter length of hospital stay compared with conventional open oesophagectomy. However, there were no significant differences in outcomes between robotic and conventional MIO techniques. No significant differences between MIO and open techniques in rates of wound or diaphragm complications, gastrointestinal complications and chyle leak were identified. Operative blood loss is difficult to measure accurately, and the clinical relevance of the small differences in operative blood loss between the surgical techniques is debatable. However, previous studies^135^, ^136^, ^137^ have suggested that volume of blood loss is an independent risk factor for postoperative adverse events, cancer recurrence and poorer overall survival. Furthermore, the potential advantages of the MIO approach, and especially the robotic approach, in decreasing operative trauma and blood loss, and improving postoperative recovery, may allow greater preservation of immune function, reduce the risk of tumour progression and allow earlier access to adjuvant treatment^138^, ^139^, ^140^, ^141^, ^142^, ^143^.

A recent meta‐analysis^18^ reported that minimally invasive approaches for oesophagectomy significantly improved long‐term survival of patients compared with conventional open surgery. However, that review did not address the impact of the different techniques on long‐term outcomes given the heterogeneity of each approach as identified by the present review. In this network meta‐analysis, TAO and MIO were only associated with a significant survival benefit compared with open surgery at 1 year, and not 3‐ or 5‐year survival. This may reflect higher rates of negative resection margins and number of lymph nodes examined with MIO and RAMIO, as identified by this review.

Based on current evidence, no single approach demonstrates clear overall superiority over all others, but there is increasing evidence of the clinical benefits of minimally invasive over open surgery.

## Supporting information


**Appendix** **S1:** Supporting information
