# Supplementary material for: Minimally invasive techniques for transthoracic oesophagectomy for oesophageal cancer: systematic review and network meta‐analysis
Source: BJS Open. 2020 Sep 7;4(5):787–803. doi: 10.1002/bjs5.50330 (PMC7528517; doi:10.1002/bjs5.50330)
Supplement: Supplementary file 1 — Appendix S1: Supporting information [file BJS5-4-787-s001.docx]

**BJS5_50330**

**Minimally invasive techniques for transthoracic oesophagectomy for oesophageal cancer: systematic review and network meta-analysis**

K. Siaw-Acheampong, S. K. Kamarajah, R. Gujjuri, J. R. Bundred, P. Singh and E. A. Griffiths

**Fig. S1** Network maps of intraoperative outcomes of overall network meta-analysis (A) Operating Time (B) Blood Loss

**Fig. S2** Network maps of postoperative complications of overall network meta-analysis (A) Overall (B) Pulmonary (C) Cardiac (D) Anastomotic Leak (E) Surgical Site infections (F) Gastrointestinal (G) Chyle Leak (H) Length of Stay (I) 30-day mortality (J) 90-day Mortality (K) 1-year Survival (L) 3-year survival (M) 5-year Survival

**Fig. S3** Network maps of oncological outcomes of overall network meta-analysis (A) Lymph Node Examined (B) R0 Resection Margin

**Table S1** Summary of search terms used

|  | **Search term** | **No of results** |
| --- | --- | --- |
| 1 | exp Oesophagectomy/ | 27929 |
| 2 | oesophagectomy.ti,ab. | 4287 |
| 3 | oesophagectomy.ti,ab. | 23044 |
| 4 | 1 or 2 or 3 | 37031 |
| 5 | exp SURVIVAL RATE/ or exp DISEASE-FREE SURVIVAL/ or exp SURVIVAL ANALYSIS/ or exp SURVIVAL/ | 1394768 |
| 6 | survival.ti,ab. | 2351353 |
| 7 | exp INTRAOPERATIVE COMPLICATIONS/ or exp POSTOPERATIVE COMPLICATIONS/ | 1154278 |
| 8 | anastomotic leaks.ab,ti. | 4665 |
| 9 | readmission.ti,ab. | 59436 |
| 10 | postoperative complications.ti,ab. | 130454 |
| 11 | margins.ti,ab. | 118219 |
| 12 | exp HOSPITAL MORTALITY/ or exp MORTALITY/ | 1380303 |
| 13 | in-hospital mortality.ti,ab. | 66867 |
| 14 | lymph nodes resected.ti,ab. | 663 |
| 15 | lymph nodes examined.ti,ab. | 1364 |
| 16 | lymph nodes harvested.ti,ab. | 1516 |
| 17 | perioperative outcomes.ti,ab. | 10687 |
| 18 | operative mortality.ti,ab. | 32855 |
| 19 | length of stay.ti,ab. | 163502 |
| 20 | Anastomotic leak.ab,ti. | 9151 |
| 21 | conversion.ti,ab. | 445044 |
| 22 | 5 or 6 or 7 or 8 or 9 or 10 or 11 or 12 or 13 or 14 or 15 or 16 or 17 or 18 or 19 or 20 or 21 | 5361196 |
| **23** | **4 and 22** | **20850** |
| 24 | exp Laparoscopy/ or exp Robotic Surgical Procedures/ or exp Minimally Invasive Surgical Procedures/ | 648165 |
| 25 | Minimally invasive.ti,ab. | 158865 |
| 26 | Ivor Lewis.ti,ab. | 1808 |
| 27 | videoThoracoscopic.ti,ab. | 1038 |
| 28 | exp Hand-Assisted Laparoscopy/ | 1017 |
| 29 | exp LAPAROTOMY/ | 89679 |
| 30 | laparotomy.ti,ab. | 113102 |
| 31 | hybrid.ti,ab. | 333715 |
| 32 | open.ti,ab. | 1251956 |
| 33 | Thoracoscopic-Laparotomy.ti,ab. | 8 |
| 34 | Thoracoscopic-Laparoscopic.ti,ab. | 179 |
| 35 | McKeown.ti,ab. | 615 |
| 36 | robot.ti,ab. | 45088 |
| 37 | da Vinci.ti,ab. | 8406 |
| 38 | robotics.ti,ab. | 12611 |
| 39 | 24 or 25 or 26 or 27 or 28 or 29 or 30 or 31 or 32 or 33 or 34 or 35 or 36 or 37 or 38 | 2389238 |
| **40** | **4 and 22 and 39** | **4961** |
| 41 | exp Esophagogastric Junction/ | 10591 |
| 42 | exp NEOPLASMS/ | 7149101 |
| 43 | 41 and 42 | 4119 |
| 44 | exp Esophageal Neoplasms/ | 120260 |
| 45 | exp Esophageal Neoplasms/ | 120260 |
| 46 | esophageal cancer.ti,ab. | 8333 |
| 47 | esophageal cancer.ti,ab. | 44983 |
| 48 | esophageal squamous cell carcinoma.ti,ab. | 17476 |
| 49 | esophageal squamous cell carcinoma.ti,ab. | 1498 |
| 50 | esophageal adenocarcinoma.ti,ab. | 10597 |
| 51 | esophageal adenocarcinoma.ti,ab. | 3111 |
| 52 | 43 or 44 or 45 or 46 or 47 or 48 or 49 or 50 or 51 | 141064 |
| **53** | **4 and 22 and 39 and 52** | **3634** |

**Table S2** Summary of intraoperative outcomes of network meta-analysis stratified by location of anastomosis

|  | **Cervical** | | | **Thoracic** | | |
| --- | --- | --- | --- | --- | --- | --- |
| **Comparison** | Studies, n | NMA, OR/MD (95% CI) | p- value | Studies, n | NMA, OR/MD (95% CI) | p- value |
| **Operating Time (minutes)** |  |  |  |  |  |  |
| Open:TAO | 8 | -17.68 ( -45.26 - 9.90) | 0.211 | 3 | -31.15 ( -96.61 - 34.30) | 0.357 |
| Open:LAO | 2 | -29.22 (-89.06 - 30.62) | 0.344 | 2 | 35.26 (- 50.40 - 120.92) | 0.428 |
| Open:MIO | 16 | -36.87 (-58.28 - -15.47) | **0.001** | 7 | -56.32 (-103.53 - - 9.12) | **0.019** |
| Open:RAMIO | 1 | -58.03 (-108.01 - -8.04) | **0.023** | 0 | -88.22 (-230.56 - 54.12) | 0.227 |
| MIO:TAO | 8 | 19.19 ( -8.45 - 46.83) | 0.174 | 2 | 25.17 ( -44.79 - 95.12) | 0.490 |
| MIO:LAO | 1 | 7.65 (-53.78 - 69.09) | 0.819 | 1 | 91.58 (- 0.25 - 183.41) | **0.050** |
| MIO :RAMIO | 3 | -21.15 ( -69.07 - 26.76) | 0.394 | 1 | -31.90 (-166.19 - 102.39) | 0.654 |
| LAO: TAO | 0 | 11.54 ( -53.35 - 76.42) | 0.740 | 1 | -66.41 (-165.12 - 32.29) | 0.188 |
| RAMIO:TAO | 0 | 40.34 ( -13.80 - 94.48) | 0.145 | 0 | 57.07 ( -94.35 - 208.48) | 0.469 |
| RAMIO:LAO | 0 | 28.81 (-47.93 - 105.54) | 0.471 | 0 | 123.48 (-39.20 - 286.16) | 0.137 |
| **Blood Loss (mls)** |  |  |  |  |  |  |
| Open:TAO | 8 | 36.21 ( -24.93 - 97.35) | 0.248 | 4 | 192.73 ( 117.60 - 267.87) | **<0.001** |
| Open:LAO | 2 | 84.29 (-37.32 - 205.89) | 0.175 | 1 | 262.19 (138.74 - 385.63) | **<0.001** |
| Open:MIO | 16 | 150.77 (108.23 - 193.31) | **<0.001** | 7 | 272.50 (222.58 - 322.41) | **<0.001** |
| Open:RAMIO | 1 | 138.78 ( 50.59 - 226.98) | **0.002** | 0 | 276.40 ( 82.63 - 470.16) | **0.005** |
| MIO:TAO | 7 | -114.56 (-176.51 - -52.61) | **<0.001** | 3 | -79.76 (-151.12 - -8.41) | **0.028** |
| MIO:LAO | 1 | -66.48 (-190.52 - 57.55) | 0.298 | 1 | -10.31 ( -137.88 - 117.26) | 0.883 |
| MIO :RAMIO | 4 | -11.99 ( -94.51 - 70.53) | 0.788 | 1 | 3.90 (-183.33 - 191.13) | 0.970 |
| LAO: TAO | 0 | -48.08 (-182.07 - 85.91) | 0.492 | 1 | -69.45 (-209.04 - 70.13) | 0.335 |
| RAMIO:TAO | 0 | -102.57 (-203.59 - -1.56) | **0.046** | 0 | -83.66 (-284.02 - 116.70) | 0.421 |
| RAMIO:LAO | 0 | -54.50 (-201.59 - 92.59) | 0.477 | 0 | -14.21 (-240.76 - 212.35) | 0.910 |

*Abbreviations: CI: Confidence Interval, LAO: Laparoscopic Assisted Oesophagectomy, MD: Mean difference, MIO: Minimally Invasive Oesophagectomy, NMA: Network Meta-Analysis, RAMIO: Robotic Minimally Invasive Oesophagectomy, TAO: Thoracoscopic Assisted Oesophagectomy*

**Table S3** Ranking of surgical techniques for intraoperative, oncological and postoperative outcomes according to P-scores

|  | **1** | **2** | **3** | **4** | **5** |
| --- | --- | --- | --- | --- | --- |
| **Operative Time** |  |  |  |  |  |
| Cervical | **Open, P = 0.9286** | TAO, P = 0.6455 | LAO, P = 0.4746 | MIO, P = 0.3243 | RAMIO, P = 0.127 |
| Thoracic | **LAO, P = 0.9007** | Open, P = 0.7281 | TAO, P = 0.4497 | MIO, P = 0.2387 | RAMIO, P = 0.1829 |
| **Blood Loss** |  |  |  |  |  |
| Cervical | MIO, P = 0.8663 | RAMIO, P = 0.7824 | LAO, P = 0.5131 | TAO, P = 0.2854 | Open, P = 0.0528 |
| Thoracic | MIO, P = 0.7581 | RAMIO, P = 0.714 | LAO, P = 0.6809 | TAO, P = 0.3464 | Open, P = 0.0007 |
| **Overall Complications** |  |  |  |  |  |
| Cervical | MIO, P = 0.6648 | LAO, P = 0.6228 | RAMIO, P = 0.555 | TAO, P = 0.4903 | Open, P = 0.1671 |
| Thoracic | RAMIO, P = 0.8323 | MIO, P = 0.6244 | LAO, P = 0.5779 | TAO, P = 0.4327 | Open, P = 0.0328 |
| **Pulmonary Complications** |  |  |  |  |  |
| Cervical | **MIO, P = 0.9404** | TAO, P = 0.6626 | RAMIO, P = 0.4539 | LAO, P = 0.2855 | Open, P = 0.1576 |
| Thoracic | MIO, P = 0.6648 | LAO, P = 0.6228 | RAMIO, P = 0.555 | TAO, P = 0.4903 | Open, P = 0.1671 |
| **Cardiac Complications** |  |  |  |  |  |
| Cervical | RAMIO, P = 0.7987 | LAO, P = 0.6864 | MIO, P = 0.6476 | Open, P = 0.2288 | TAO, P = 0.1386 |
| Thoracic | TAO, P = 0.7555 | Open, P = 0.5632 | MIO, P = 0.4884 | LAO, P = 0.1929 |  |
| **Anastomotic Leak** |  |  |  |  |  |
| Cervical | RAMIO, P = 0.80 | LAO, P = 0.6864 | MIO, P = 0.6476 | Open, P = 0.2288 | TAO, P = 0.1386 |
| Thoracic | TAO, P = 0.76 | Open, P = 0.5632 | MIO, P = 0.4884 | LAO, P = 0.1929 |  |
| **Surgical site infections** |  |  |  |  |  |
| Cervical | TAO, P = 0.8847 | RAMIO, P = 0.6609 | Open, P = 0.4338 | MIO, P = 0.2949 | LAO, P = 0.2256 |
| Thoracic | TAO, P = 0.7268 | LAO, P = 0.5867 | MIO, P = 0.4763 | Open, P = 0.2102 |  |
| **Gastrointestinal Complications** |  |  |  |  |  |
| Cervical | MIO, P = 0.8526 | TAO, P = 0.7796 | RAMIO, P = 0.3512 | Open, P = 0.3325 | LAO, P = 0.184 |
| Thoracic | TAO, P = 0.7346 | Open, P = 0.6868 | MIO, P = 0.5727 | LAO, P = 0.3426 | RAMIO, P = 0.1635 |
| **Chyle Leak** |  |  |  |  |  |
| Cervical | RAMIO, P = 0.7182 | MIO, P = 0.6247 | LAO, P = 0.4345 | Open, P = 0.3697 | TAO, P = 0.3529 |
| Thoracic | TAO, P = 0.8008 | Open, P = 0.5928 | MIO, P = 0.3714 | LAO, P = 0.235 |  |
| **LOS** |  |  |  |  |  |
| Cervical | **MIO, P = 0.9122** | RAMIO, P = 0.6698 | TAO, P = 0.5545 | LAO, P = 0.2094 | Open, P = 0.1541 |
| Thoracic | MIO, P = 0.7276 | TAO, P = 0.6272 | LAO, P = 0.5617 | Open, P = 0.0836 |  |
| **30-day mortality** |  |  |  |  |  |
| Cervical | RAMIO, P = 0.8008 | MIO, P = 0.6056 | TAO, P = 0.4687 | Open, P = 0.1248 |  |
| Thoracic | Open, P = 0.8254 | MIO, P = 0.5836 | RAMIO, P = 0.4385 | TAO, P = 0.1524 |  |
| **90-day mortality** |  |  |  |  |  |
| Cervical | MIO, P = 0.7025 | RAMIO, P = 0.6756 | Open, P = 0.1219 |  |  |
| Thoracic | **MIO, P = 0.9344** | Open, P = 0.0656 |  |  |  |
| **Lymph Nodes** |  |  |  |  |  |
| Cervical | **LAO, P = 0.9391** | TAO, P = 0.5352 | Open, P = 0.4356 | MIO, P = 0.4011 | RAMIO, P = 0.1891 |
| Thoracic | LAO, P = 0.8712 | Open, P = 0.7245 | TAO, P = 0.5928 | MIO, P = 0.288 | RAMIO, P = 0.0234 |
| **R0 Margins** |  |  |  |  |  |
| Cervical | RAMIO, P = 0.7102 | Open, P = 0.6689 | TAO, P = 0.5155 | LAO, P = 0.3273 | MIO, P = 0.2781 |
| Thoracic | **Open, P = 0.9852** | MIO, P = 0.3573 | TAO, P = 0.1575 |  |  |
| **1-year Survival** |  |  |  |  |  |
| Cervical | MIO, P = 0.6847 | TAO, P = 0.6148 | Open, P = 0.5174 | RAMIO, P = 0.1832 |  |
| Thoracic | LAO, P = 0.8212 | TAO, P = 0.721 | MIO, P = 0.3236 | Open, P = 0.1342 |  |
| **3-year Survival** |  |  |  |  |  |
| Cervical | RAMIO, P = 0.6371 | TAO, P = 0.5629 | Open, P = 0.4807 | MIO, P = 0.3193 |  |
| Thoracic | **LAO, P = 0.9287** | MIO, P = 0.3765 | Open, P = 0.3739 | TAO, P = 0.3208 |  |
| **5-year Survival** |  |  |  |  |  |
| Cervical | RAMIO, P = 0.8732 | TAO, P = 0.4925 | MIO, P = 0.4168 | Open, P = 0.2175 |  |
| Thoracic | TAO, P = 0.5674 | MIO, P = 0.5094 | LAO, P = 0.5093 | Open, P = 0.4138 |  |

**Table S4** Summary of postoperative outcomes of network meta-analysis stratified by location of anastomosis

|  | **Cervical** | | | **Thoracic** | | |
| --- | --- | --- | --- | --- | --- | --- |
| **Comparison** | Studies, n | NMA, OR (95% CI) | p- value | Studies, n | NMA, OR (95% CI) | p- value |
| **Overall Complications** |  |  |  |  |  |  |
| Open:TAO | 3 | 1.52 (0.96 - 2.41) | 0.074 | 3 | 1.52 (0.96 - 2.41) | 0.074 |
| Open:LAO | 1 | 1.75 (0.79 - 4.00) | 0.176 | 1 | 1.75 (0.79 - 4) | 0.176 |
| Open:MIO | 6 | 1.79 (1.28: 2.5) | **0.001** | 6 | 1.79 (1.28: 2.5) | **0.001** |
| Open:RAMIO | 1 | 2.38 (1.17 - 4.84) | **0.017** | 1 | 2.38 (1.17 - 4.84) | **0.017** |
| MIO:TAO | 2 | 0.85 (0.53 - 1.35) | 0.506 | 2 | 0.85 (0.53 - 1.35) | 0.506 |
| MIO:LAO | 0 | 0.98 (0.41 - 2.33) | 0.968 | 0 | 0.98 (0.41 - 2.33) | 0.968 |
| MIO :RAMIO | 1 | 1.33 (0.63 - 2.78) | 0.460 | 1 | 1.33 (0.63 - 2.78) | 0.460 |
| LAO: TAO | 0 | 0.86 (0.34 - 2.18) | 0.763 | 0 | 0.86 (0.34 - 2.18) | 0.763 |
| RAMIO:TAO | 0 | 0.64 (0.28 - 1.46) | 0.293 | 0 | 0.64 (0.28 - 1.46) | 0.293 |
| RAMIO:LAO | 0 | 0.74 (0.25 - 2.17) | 0.596 | 0 | 0.74 (0.25 - 2.17) | 0.596 |
| **Pulmonary Comps** |  |  |  |  |  |  |
| Open:TAO | 7 | 1.87 (1.19 - 2.96) | **0.007** | 4 | 1.47 (0.69 - 3.14) | 0.324 |
| Open:LAO | 2 | 1.09 (0.38 - 3.13) | 0.886 | 2 | 1.92 (0.40 - 9.09) | 0.420 |
| Open:MIO | 14 | 2.56 (1.79 - 3.57) | **<0.001** | 8 | 1.79 (1.04 - 3.13) | **0.038** |
| Open:RAMIO | 1 | 1.43 (0.64 - 3.22) | 0.392 | 0 | 1.72 (0.27 - 11.04) | 0.579 |
| MIO:TAO | 6 | 0.74 (0.46 - 1.18) | 0.212 | 3 | 0.82 (0.37 - 1.81) | 0.637 |
| MIO:LAO | 1 | 0.43 (0.15 - 1.27) | 0.124 | 1 | 1.08 (0.21 - 5.56) | 0.937 |
| MIO :RAMIO | 4 | 0.56 (0.26 - 1.22) | 0.142 | 1 | 0.95 (0.16 - 5.65) | 0.959 |
| LAO: TAO | 0 | 1.72 (0.55 - 5.32) | 0.355 | 1 | 0.76 (0.14 - 4.16) | 0.764 |
| RAMIO:TAO | 0 | 1.31 (0.54 - 3.16) | 0.561 | 0 | 0.85 (0.12 - 6.00) | 0.880 |
| RAMIO:LAO | 0 | 0.76 (0.21 - 2.78) | 0.697 | 0 | 1.12 (0.10 - 12.50) | 0.931 |
| **Cardiac Comps** |  |  |  |  |  |  |
| Open:TAO | 2 | 0.87 (0.45 - 1.67) | 0.690 | 3 | 1.25 (0.55 - 2.82) | 0.605 |
| Open:LAO | 1 | 2.08 (0.50 - 9.09) | 0.328 | 1 | 0.47 (0.08 - 2.78) | 0.411 |
| Open:MIO | 7 | 1.64 (1.03 - 2.63) | **0.038** | 5 | 0.96 (0.71 - 1.32) | 0.815 |
| Open:RAMIO | 1 | 2.59 (0.71 - 9.48) | 0.150 |  |  |  |
| MIO:TAO | 3 | 0.53 (0.30 - 0.94) | **0.029** | 2 | 1.29 (0.57 - 2.95) | 0.555 |
| MIO:LAO | 0 | 1.28 (0.28 - 5.88) | 0.762 | 0 | 0.49 (0.08 - 2.94) | 0.439 |
| MIO :RAMIO | 2 | 1.58 (0.42 - 5.94) | 0.509 |  |  |  |
| LAO: TAO | 0 | 0.41 (0.08 - 2.02) | 0.283 | 0 | 2.66 (0.38 - 18.64) | 0.330 |
| RAMIO:TAO | 0 | 0.34 (0.08 - 1.38) | 0.138 |  |  |  |
| RAMIO:LAO | 0 | 0.81 (0.12 - 5.56) | 0.845 |  |  |  |
| **Anastomotic Leaks** |  |  |  |  |  |  |
| Open:TAO | 2 | 0.87 (0.45 - 1.67) | 0.690 | 3 | 1.25 (0.55 - 2.82) | 0.605 |
| Open:LAO | 1 | 2.08 (0.50 - 9.09) | 0.328 | 1 | 0.47 (0.08 - 2.77) | 0.411 |
| Open:MIO | 7 | 1.64 (1.03 - 2.63) | **0.038** | 5 | 0.96 (0.71 - 1.32) | 0.815 |
| Open:RAMIO | 1 | 2.59 (0.71 - 9.48) | 0.150 |  |  |  |
| MIO:TAO | 3 | 0.53 (0.30 - 0.94) | **0.029** | 2 | 1.29 (0.57 - 2.95) | 0.555 |
| MIO:LAO | 0 | 1.28 (0.28 - 5.88) | 0.762 | 0 | 0.49 (0.08: 2.94) | 0.439 |
| MIO :RAMIO | 2 | 1.58 (0.42 - 5.94) | 0.509 |  |  |  |
| LAO: TAO | 0 | 0.41 (0.08 - 2.02) | 0.283 | 0 | 2.66 (0.38 - 18.64) | 0.330 |
| RAMIO:TAO | 0 | 0.34 (0.08 - 1.38) | 0.138 |  |  |  |
| RAMIO:LAO | 0 | 0.81 (0.12 - 5.56) | 0.845 |  |  |  |
| **Surgical site infections** |  |  |  |  |  |  |
| Open:TAO | 1 | 6.09 (0.82 - 45.06) | 0.077 | 0 | 5.73 (0.16 - 204.78) | 0.344 |
| Open:LAO | 5 | 0.70 (0.23 - 2.17) | 0.536 | 1 | 3.13 (0.12 - 100) | 0.516 |
| Open:MIO | 12 | 0.83 (0.43 - 1.64) | 0.559 | 2 | 1.89 (0.39 - 9.09) | 0.436 |
| Open:RAMIO | 1 | 3.00 (0.10 - 94.13) | 0.540 |  |  |  |
| MIO:TAO | 1 | 7.29 (0.95 - 56.01) | 0.056 | 1 | 3.04 (0.12 - 75.69) | 0.509 |
| MIO:LAO | 2 | 0.84 (0.27 - 2.63) | 0.754 | 0 | 1.64 (0.05 - 50) | 0.795 |
| MIO :RAMIO | 0 | 3.59 (0.11 - 120.31) | 0.483 |  |  |  |
| LAO: TAO | 0 | 8.66 (0.90 - 83.54) | 0.061 | 0 | 1.85 (0.01 - 230.50) | 0.822 |
| RAMIO:TAO | 0 | 2.03 (0.04 - 109.19) | 0.739 |  |  |  |
| RAMIO:LAO | 0 | 0.23 (0.01 - 9.09) | 0.444 |  |  |  |
| **GI Complications** |  |  |  |  |  |  |
| Open:TAO | 6 | 1.68 (0.95 - 2.97) | 0.074 | 4 | 1.07 (0.56 - 2.05) | 0.849 |
| Open:LAO | 2 | 0.71 (0.26 - 1.96) | 0.524 | 2 | 0.55 (0.13 - 2.33) | 0.424 |
| Open:MIO | 15 | 1.79 (1.20 - 2.63) | **0.004** | 9 | 0.93 (0.63 - 1.35) | 0.704 |
| Open:RAMIO | 1 | 0.93 (0.27 - 3.15) | 0.915 | 0 | 0.25 (0.02 - 2.54) | 0.265 |
| MIO:TAO | 7 | 0.94 (0.55 - 1.61) | 0.833 | 3 | 1.16 (0.61 - 2.20) | 0.663 |
| MIO:LAO | 1 | 0.40 (0.14 - 1.14) | 0.084 | 1 | 0.60 (0.14 - 2.63) | 0.503 |
| MIO :RAMIO | 4 | 0.52 (0.16 - 1.69) | 0.280 | 1 | 0.27 (0.03 - 2.66) | 0.255 |
| LAO: TAO | 0 | 2.36 (0.76 - 7.34) | 0.138 | 1 | 1.95 (0.41 - 9.32) | 0.409 |
| RAMIO:TAO | 0 | 1.81 (0.50 - 6.57) | 0.373 | 0 | 4.27 (0.40 - 45.80) | 0.232 |
| RAMIO:LAO | 0 | 0.77 (0.16 - 3.70) | 0.756 | 0 | 2.17 (0.14 - 33.33) | 0.588 |
| **Chyle Leak** |  |  |  |  |  |  |
| Open:TAO | 4 | 0.96 (0.41 - 2.28) | 0.930 | 1 | 1.87 (0.33 - 10.55) | 0.489 |
| Open:LAO | 1 | 1.02 (0.14 - 7.14) | 0.985 | 1 | 0.32 (0.01 - 8.33) | 0.505 |
| Open:MIO | 7 | 1.33 (0.67 - 2.63) | 0.394 | 3 | 0.84 (0.55 - 1.28) | 0.429 |
| Open:RAMIO | 0 | 2.17 (0.23 - 20.18) | 0.507 |  |  |  |
| MIO:TAO | 3 | 0.72 (0.31 - 1.67) | 0.441 | 1 | 2.22 (0.39 - 12.54) | 0.374 |
| MIO:LAO | 0 | 0.76 (0.09 - 6.25) | 0.813 | 0 | 0.38 (0.01 - 10) | 0.578 |
| MIO :RAMIO | 2 | 1.63 (0.19 - 13.57) | 0.666 |  |  |  |
| LAO: TAO | 0 | 0.95 (0.11 - 8.18) | 0.966 | 0 | 5.77 (0.15 - 226.50) | 0.354 |
| RAMIO:TAO | 0 | 0.44 (0.05 - 4.35) | 0.479 |  |  |  |
| RAMIO:LAO | 0 | 0.47 (0.02 - 9.09) | 0.631 |  |  |  |
| **LOS** |  |  |  |  |  |  |
| Open:TAO | 6 | 2.52 (-0.38 - 5.42) | 0.088 | 3 | 2.48 (-1.07 - 6.03) | 0.172 |
| Open:LAO | 2 | 7.14 (-0.20 - 0.19) | 0.961 | 2 | 0.47 (0.16 - 0.53) | 0.300 |
| Open:MIO | 15 | -0.221/ (-0.38 - -0.15) | **<0.001** | 7 | -0.35 (-4.55 - -0.18) | **0.033** |
| Open:RAMIO | 1 | 3.30 (-0.64 - 7.23) | 0.100 |  |  |  |
| MIO:TAO | 7 | -2.11 (-4.97 - 0.75) | 0.149 | 3 | -0.37 (-3.88 - 3.13) | 0.847 |
| MIO:LAO | 1 | 0.21 (-2.27 - 0.10) | 0.072 | 1 | 1.43 (-0.25 - 0.19) | 0.781 |
| MIO :RAMIO | 3 | -1.33 (-5.04 - 2.37) | 0.491 |  |  |  |
| LAO: TAO | 0 | 2.66 (-3.07 - 8.40) | 0.369 | 1 | 0.33 (-4.77 - 5.43) | 0.907 |
| RAMIO:TAO | 0 | -0.78 (-5.35 - 3.80) | 0.751 |  |  |  |
| RAMIO:LAO | 0 | 0.29 (-0.35 - 0.10) | 0.287 |  |  |  |
| **30-day mortality** |  |  |  |  |  |  |
| Open:TAO | 0 | 2.05 (0.43 - 9.77) | 0.361 | 1 | 0.09 (0.00 - 2.36) |  |
| Open:LAO |  |  |  |  |  |  |
| Open:MIO | 4 | 2.44 (0.76 - 7.69) | 0.086 | 6 | 0.77 (0.44 - 1.35) | 0.365 |
| Open:RAMIO | 0 | 7.55 (0.24 - 234.22) | 0.252 | 0 | 0.36 (0.01 - 9.55) | 0.571 |
| MIO:TAO | 1 | 0.83 (0.30 - 2.34) | 0.713 | 0 | 0.12 (0.00 - 3.21) | 0.226 |
| MIO:LAO |  |  |  |  |  |  |
| MIO :RAMIO | 1 | 3.07 (0.12 - 77.59) | 0.507 | 1 | 0.47 (0.02 - 11.84) | 0.656 |
| LAO: TAO |  |  |  |  |  |  |
| RAMIO:TAO | 0 | 0.27 (0.01 - 8.05) | 0.451 | 0 | 0.25 (0.00 - 25.43) | 0.604 |
| RAMIO:LAO |  |  |  |  |  |  |
| **90-day mortality** |  |  |  |  |  |  |
| Open:TAO |  |  |  |  |  |  |
| Open:LAO |  |  |  |  |  |  |
| Open:MIO | 1 | 7.14 (0.37 - 100) | 0.146 | 1 | 1.81 (0.84 - 3.65) | 0.123 |
| Open:RAMIO | 0 | 7.38 (0.17 - 317.86) | 0.302 |  |  |  |
| MIO:TAO |  |  |  |  |  |  |
| MIO:LAO |  |  |  |  |  |  |
| MIO :RAMIO | 2 | 1.00 (0.10 - 9.89) | 1.000 |  |  |  |
| LAO: TAO |  |  |  |  |  |  |
| RAMIO:TAO |  |  |  |  |  |  |
| RAMIO:LAO |  |  |  |  |  |  |
| **1-year Survival** |  |  |  |  |  |  |
| Open:TAO | 2 | 1.58 (0.80 - 3.14) | 0.191 | 3 | 1.12 (0.38 - 3.28) | 0.848 |
| Open:LAO | 2 | 1.69 (0.99 - 2.94) | 0.057 |  |  |  |
| Open:MIO | 9 | 1.10 (0.79 - 1.52) | 0.579 | 6 | 1.19 (0.54 - 2.63) | 0.679 |
| Open:RAMIO |  |  |  | 0 | 0.34 (0.03 - 4.26) | 0.401 |
| MIO:TAO | 0 | 1.44 (0.67 - 3.06) | 0.352 | 1 | 0.94 (0.28 - 3.14) | 0.927 |
| MIO:LAO | 1 | 1.54 (0.85 - 2.78) | 0.152 |  |  |  |
| MIO :RAMIO |  |  |  | 1 | 0.29 (0.03 - 3.15) | 0.301 |
| LAO: TAO | 0 | 0.93 (0.39 - 2.22) | 0.879 |  |  |  |
| RAMIO:TAO | 2 | 1.58 (0.80 - 3.14) | 0.191 | 0 | 3.27 (0.22 - 47.81) | 0.395 |
| RAMIO:LAO | 2 | 1.69 (0.99 - 2.94) | 0.057 |  |  |  |
| **3-year Survival** |  |  |  |  |  |  |
| Open:TAO | 2 | 0.90 (0.30 - 2.67) | 0.860 | 2 | 1.29 (0.14 - 11.77) | 0.833 |
| Open:LAO | 2 | 2.13 (0.88 - 5.26) | 0.097 |  |  |  |
| Open:MIO | 8 | 1.00 (0.61 - 1.64) | 1.000 | 5 | 0.74 (0.18 - 2.94) | 0.678 |
| Open:RAMIO |  |  |  | 0 | 1.82 (0.06 - 57.12) | 0.745 |
| MIO:TAO | 0 | 0.90 (0.27 - 2.97) | 0.873 | 0 | 1.75 (0.13 - 23.96) | 0.687 |
| MIO:LAO | 1 | 2.13 (0.84 - 5.56) | 0.117 |  |  |  |
| MIO :RAMIO |  |  |  | 1 | 2.47 (0.11 - 57.62) | 0.583 |
| LAO: TAO | 0 | 0.42 (0.10 - 1.70) | 0.232 |  |  |  |
| RAMIO:TAO | 2 | 0.90 (0.30 - 2.67) | 0.860 | 0 | 0.71 (0.01 - 42.59) | 0.882 |
| RAMIO:LAO | 2 | 2.13 (0.88 - 5.26) | 0.097 |  |  |  |
| **5-year Survival** |  |  |  |  |  |  |
| Open:TAO | 1 | 1.42 (0.10 - 20.24) | 0.808 | 2 | 1.43 (0.44 - 4.60) | 0.562 |
| Open:LAO | 1 | 1.18 (0.13 - 11.11) | 0.895 |  |  |  |
| Open:MIO | 6 | 1.15 (0.38 - 3.45) | 0.815 | 4 | 1.28 (0.53 - 3.03) | 0.588 |
| Open:RAMIO |  |  |  | 0 | 3.83 (0.57 - 25.84) | 0.168 |
| MIO:TAO | 0 | 1.24 (0.07 - 21.93) | 0.892 | 0 | 1.12 (0.26 - 4.82) | 0.888 |
| MIO:LAO | 1 | 1.03 (0.11 - 9.09) | 0.980 |  |  |  |
| MIO :RAMIO |  |  |  | 1 | 3.00 (0.55 - 16.38) | 0.206 |
| LAO: TAO | 0 | 1.20 (0.04 - 38.18) | 0.924 |  |  |  |
| RAMIO:TAO | 1 | 1.42 (0.10 - 20.24) | 0.808 | 0 | 0.37 (0.04 - 3.50) | 0.390 |
| RAMIO:LAO | 1 | 1.18 (0.13 - 11.11) | 0.895 |  |  |  |

**Table S5** Summary of oncological outcomes of network meta-analysis stratified by location of anastomosis

|  | **Cervical** | | | **Thoracic** | | |
| --- | --- | --- | --- | --- | --- | --- |
| **Comparison** | Studies, n | NMA, OR/MD (95% CI) | p- value | Studies, n | NMA, OR/MD (95% CI) | p- value |
| **Lymph Node Examined** |  |  |  |  |  |  |
| Open:TAO | 7 | 0.35 ( -1.98 - 2.67) | 0.781 | 4 | -0.96 ( -5.32 - 3.40) | 0.679 |
| Open:LAO | 2 | -0.26 ( -0.12 - 1.41) | 0.097 | 2 | -0.45 ( -0.12 - 0.24) | 0.511 |
| Open:MIO | 13 | 7.69 ( -0.59 - 0.51) | 0.898 | 10 | 0.29 (0.16 - 1.43) | **0.014** |
| Open:RAMIO | 0 | -1.55 ( -6.01 - 2.90) | 0.505 | 0 | -12.05 (-22.48 - -1.62) | 0.023 |
| MIO:TAO | 6 | 0.48 ( -1.92 - 2.87) | 0.708 | 3 | 2.49 ( -2.03 - 7.01) | 0.284 |
| MIO:LAO | 0 | -0.25 ( -0.11 - 1.08) | 0.112 | 1 | -0.18 (-0.08 - 0.87) | 0.103 |
| MIO :RAMIO | 4 | -1.42 ( -5.49 - 2.64) | 0.504 | 1 | -8.60 (-18.66 - 1.46) | 0.094 |
| LAO: TAO | 0 | -3.49 ( -8.60 - 1.61) | 0.181 | 1 | -3.17 (-10.62 - 4.28) | 0.412 |
| RAMIO:TAO | 0 | 1.90 ( -2.82 - 6.62) | 0.438 | 0 | 11.09 ( 0.06 - 22.12) | **0.048** |
| RAMIO:LAO | 0 | -0.19 (-0.09 - 1.03) | 0.097 | 0 | -0.07 (-0.47 - -0.04) | **0.021** |
| **R0 Margins** |  |  |  |  |  |  |
| Open:TAO | 2 | 0.81 (0.34 - 1.92) | 0.453 | 2 | 0.59 (0.35 - 0.99) | **0.046** |
| Open:LAO | 1 | 0.49 (0.04 - 5.56) | 0.571 |  |  |  |
| Open:MIO | 2 | 0.62 (0.32 - 1.20) | 0.141 | 7 | 0.68 (0.51 - 0.93) | **0.014** |
| Open:RAMIO | 0 | 1.91 (0.07 - 51.99) | 0.714 |  |  |  |
| MIO:TAO | 2 | 1.31 (0.68 - 2.53) | 0.332 | 2 | 0.86 (0.49 - 1.49) | 0.607 |
| MIO:LAO | 0 | 0.79 (0.06 - 10.00) | 0.862 |  |  |  |
| MIO :RAMIO | 1 | 3.09 (0.12 - 78.55) | 0.505 |  |  |  |
| LAO: TAO | 0 | 1.67 (0.13 - 21.68) | 0.706 |  |  |  |
| RAMIO:TAO | 0 | 0.42 (0.02 - 11.52) | 0.602 |  |  |  |
| RAMIO:LAO | 0 | 0.25 (0.00 - 14.29) | 0.519 |  |  |  |
